# Genetic variants in melanogenesis proteins *TYRP1* and *TYR* are associated with the golden rhesus macaque phenotype

**DOI:** 10.1093/g3journal/jkad168

**Published:** 2023-07-31

**Authors:** Samuel M Peterson, Marina M Watowich, Lauren M Renner, Samantha Martin, Emma Offenberg, Amanda Lea, Michael J Montague, James P Higham, Noah Snyder-Mackler, Martha Neuringer, Betsy Ferguson

**Affiliations:** Division of Genetics, Oregon National Primate Research Center, Beaverton, OR 97006, USA; Department of Biology, University of Washington, Seattle, WA 98195, USA; Center for Evolution and Medicine, Arizona State University, Tempe, AZ 85281, USA; Department of Biological Sciences, Vanderbilt University, Nashville, TN 37235, USA; Division of Neuroscience, Oregon National Primate Research Center, Beaverton, OR 97006, USA; Division of Genetics, Oregon National Primate Research Center, Beaverton, OR 97006, USA; Center for Evolution and Medicine, Arizona State University, Tempe, AZ 85281, USA; Department of Biological Sciences, Vanderbilt University, Nashville, TN 37235, USA; Child and Brain Development Program, Canadian Institute for Advanced Research, Toronto, ON M5G 1M1, Canada; Department of Neuroscience, Perelman School of Medicine, University of Pennsylvania, Philadelphia, PA 19104, USA; Department of Anthropology, New York University, New York, NY 10003, USA; Center for Evolution and Medicine, Arizona State University, Tempe, AZ 85281, USA; School of Life Sciences, Arizona State University, Tempe, AZ 85281, USA; School for Human Evolution & Social Change, Arizona State University, Tempe, AZ 85281, USA; Division of Neuroscience, Oregon National Primate Research Center, Beaverton, OR 97006, USA; Casey Eye Institute, Oregon Health & Science University, Portland, OR 97239, USA; Division of Genetics, Oregon National Primate Research Center, Beaverton, OR 97006, USA; Division of Neuroscience, Oregon National Primate Research Center, Beaverton, OR 97006, USA

**Keywords:** nonhuman primate, TYRP1, TYR, rare disease, genetic, Genetics Models of Rare Diseases

## Abstract

Nonhuman primates (NHPs) are vital translational research models due to their high genetic, physiological, and anatomical homology with humans. The “golden” rhesus macaque (*Macaca mulatta*) phenotype is a naturally occurring, inherited trait with a visually distinct pigmentation pattern resulting in light blonde colored fur. Retinal imaging also reveals consistent hypopigmentation and occasional foveal hypoplasia. Here, we describe the use of genome-wide association in 2 distinct NHP populations to identify candidate variants in genes linked to the golden phenotype. Two missense variants were identified in the *Tyrosinase-related protein 1* gene (Asp343Gly and Leu415Pro) that segregate with the phenotype. An additional and distinct association was also found with a *Tyrosinase* variant (His256Gln), indicating the light-colored fur phenotype can result from multiple genetic mechanisms. The implicated genes are related through their contribution to the melanogenesis pathway. Variants in these 2 genes are known to cause pigmentation phenotypes in other species and to be associated with oculocutaneous albinism in humans. The novel associations presented in this study will permit further investigations into the role these proteins and variants play in the melanogenesis pathway and model the effects of genetic hypopigmentation and altered melanogenesis in a naturally occurring nonhuman primate model.

## Teaser

Peterson et al. describe the first genetic mapping of the “golden” rhesus macaque phenotype, a naturally occurring pigmentation trait. Using independent analyses of macaques at two different US Primate Research Centers the authors were able to attribute the phenotype to 3 different variants in the *TYRP1* and *TYR* genes. They highlight the use of the macaque Genotype and Phenotype (mGAP) Resource to identify the genetic basis for the trait and its applicability as a non-human primate genetic model for oculocutaneous albinism.

## Introduction

The great diversity of mammalian coloration has served as the basis for many genetic and evolutionary investigations into environmental selective pressures and genotype-to-phenotype links (Bradley and Mundy 2008; Caro *et al*. 2021). Hundreds of genes and pathways are capable of affecting color in vertebrates (Hoekstra 2006), but melanin-derived pigmentation serves as the most prominent driver of mammalian color diversity. Melanogenesis occurs in the highly specialized melanocyte cells, which convert the amino acid tyrosine into either eumelanin (dark brown to black) or pheomelanin (yellow to red) pigments through a series of enzymatic reactions. Controlled by intricate signaling processes, the complexity of observed coloration and color patterning results from different spatiotemporal melanocyte localization, activity levels, and the ratio of eumelanin/pheomelanin synthesis (Caro and Mallarino 2020). A subset of genes relating to melanocyte signaling or enzymatic activity are noteworthy in the context of human health, due to their association with hypopigmentation and their clinical significance in the pathogenesis of oculocutaneous albinism (OCA; Fernández *et al*. 2021).

A complex genetic architecture drives coloration within and among species and is the result of a large number of genetic variants interacting throughout development (San-Jose and Roulin 2017). Regardless, the melanogenesis pathway remains conserved in vertebrates and many simple Mendelian traits have been attributed to mutations in a small number of melanogenesis-specific genes. Often, these traits have adaptive characteristics, such as the *Agouti-*driven cryptic coloration of deer mice (Barrett *et al*. 2019) or the aposematic coloration of venomous slow lorises (Munds *et al*. 2021). Alternatively, there are examples of natural pigmentation phenotypes that can result in a loss of fitness. Although rare in nature due to strong negative selection pressures, cases of albinism have been observed in many species, including nonhuman primates (NHPs), such as a Western lowland gorilla, associated with a missense variant in *SLC45A2* (Prado-Martinez *et al*. 2013), and an albino chimpanzee that was not genetically characterized (Leroux *et al*. 2022). Numerous classic coloration morphs under artificial selection pressure in domestic pets and economic livestock have also been linked to these same melanogenesis genes (Schmutz and Berryere 2007; Ren *et al.* 2011).

In humans, variations in melanogenesis-related genes are associated with OCA, a polygenic autosomal recessive disorder characterized by the absence or reduced presence of melanin, in which individuals have pale skin, light-colored hair, and eyes (Summers 2009; Marçon and Maia 2019), and an increase in skin cancer risk (Okulicz *et al*. 2003; Mabula *et al*. 2012) or retinal associated pathologies such as nystagmus, photophobia, or foveal hypoplasia (Summers 2009; Rocca *et al*. 2022). At least 8 subtypes of OCA have been characterized (see review Ullah 2022) distinguished by the affected gene, resulting in a wide clinical spectrum, ranging from a complete lack of melanin in severe cases of OCA1 to mild, age-related hypopigmentation observed in other OCA subtypes. Genetic models for OCA in classic laboratory animals (Onojafe *et al*. 2018; Zhang *et al*. 2019; Winkler *et al*. 2014; Neuffer and Cooper 2022) recapitulate the hypopigmentation characteristic of the disease, supporting study of melanogenesis as well as some aspects of OCA pathogenesis. However, these models are less informative for the analysis of retinal effects, since only primate retinal structures include a macula and fovea (Bringmann *et al*. 2018). NHP models of altered melanogenesis with genetic homology to OCA have a significant role in advancing the study of melanin in development, disease pathogenesis, and preclinical therapeutic trials (Slijkerman *et al*. 2015; Duncan *et al*. 2018). NHP models of OCA have been reported (Scheepers 1939; Fooden 1979; Mahabal *et al*. 2012; Espinal *et al*. 2016; Zhongming *et al*. 2018; Leroux *et al*. 2022), with animals frequently having an albino appearance. But in most cases, the genetic basis was not determined, and the often-small number of individuals, largely in wild populations, has limited their utility for further study.

Herein, we report the genetic basis of naturally occurring hypopigmentation traits in rhesus macaques, previously characterized in a free-ranging colony on the island of Cayo Santiago, Puerto Rico (Kessler and Rawlins 2016; Rawlins and Kessler 1983; Kessler and Kaufman 1986) and observed at several National Primate Research Centers (NPRCs). Genetic linkage was determined through independent analyses of separate macaque populations, substantiating the shared findings. Affected macaques, colloquially referred to as “golden” or “blond,” have lighter-colored fur than other rhesus but maintain the typical 2-toned body pattern of the species. We determined the golden phenotype is caused by missense variants in either of 2 genes, *TYRP1* or *TYR*; genes associated with human OCA3 and OCA1B. Both golden traits present with similar pelage and are inherited in an autosomal recessive fashion. Despite the genetic similarity to human OCA, we note only partial recapitulation with the human disease in that the prominently altered fur covers skin that is naturally pale and thus, not notably affected. Regardless, golden rhesus macaques represent a valuable resource for studying the effect of coloration in a controlled NHP population and the functional role of melanogenesis and OCA-related genes in primates. The mapping of the genetic basis for the trait will allow for more advanced characterization, genetic management, and delineation of genotype-to-phenotype-related differences.

This genetic analysis was aided by the publicly available macaque genotype and phenotype resource (mGAP; https://mgap.ohsu.edu/; Bimber *et al*. 2019) which serves as a powerful repository of genomic variants observed in rhesus macaques across multiple NPRCs and study cohorts with relevant data annotations including observed allele frequency, predicted gene function impact, and evolutionary conservation scores. By providing naturally occurring variant data across rhesus populations, and including catalogs of rare and potentially impactful variants, the mGAP database, as well as other similar resources (Rogers 2022), support and strengthen genetic studies aiming to identify genotype-to-phenotype correlations or discover preclinical NHP disease models (Bimber *et al*. 2017).

## Materials and methods

### Animals

All animal research was conducted at the Oregon National Primate Research Center (ONPRC) at Oregon Health & Science University (OHSU) and on Cayo Santiago, operated by the Caribbean Primate Research Center (CPRC) at the University of Puerto Rico; both of which are accredited by the Association for Assessment and Accreditation of Laboratory Animal Care, International. For animals at the ONPRC, animal care and procedures were approved by the OHSU Institutional Animal Care and Use Committee and were in compliance with the *Guide for the Care and Use of Laboratory Animals* (National Research Council 2011). All work with Cayo Santiago animals was reviewed and approved by Institutional Animal Care and Use Committees of Arizona State University and the University of Puerto Rico, Medical Sciences Campus. The ONPRC is home to over 4,500 rhesus macaques, members of a molecularly pedigreed colony having detailed, electronic health records that date back several generations. Rhesus macaques included in this study are of Indian-origin ancestry. The golden phenotype was identified in individuals through direct observation or notations in electronic health records.

### DNA extraction and genotyping

Genomic DNA from ONPRC-housed rhesus macaques was accessed through the ONPRC Genetic Core DNA Bank or extracted from archived tissue samples using a Maxwell RSC Instrument and following the manufacturer's protocol (Promega, Inc.). Genotyping-by-sequencing (GBS) libraries were produced as previously described (Bimber *et al*. 2016) using the *PSTI* restriction enzyme and sequenced on an Illumina NextSeq500 at the Oregon Health & Science University Massively Parallel Sequencing Shared Resource or an Illumina HiSeq 4000 at the Genomics & Cell Characterization Core Facility at University of Oregon. For macaques from Cayo Santiago, 6 ml of peripheral whole blood was drawn by veterinary staff into EDTA tubes and frozen for long-term storage. Following DNA extraction, whole-genome sequencing (WGS) libraries were generated using the Nextera DNA library preparation kit and sequenced using paired-end sequencing on the Illumina NovaSeq platform.

The mGAP database (Bimber *et al*. 2019) was used to access genetic variant annotations including population-specific allele frequencies, predicted gene impact, pedigree information, and pathogenicity prediction scores from the Combined Annotation Dependent Depletion (CADD; Rentzsch *et al*. 2021), Polymorphism Phenotyping v2 (PolyPhen; Adzhubei *et al*. 2010), and Sorting Intolerant From Tolerant (SIFT; Sim *et al*. 2012) tools. Additional genotyping was performed following PCR amplification of the genomic region of interest using primers *TYRP1_*Ex*5*_F/R: GCAAGTGGTTAAAAATATCATCCT/TTGGACAAATTGTTTCCCAATA or *TYRP1*_Ex4_F/R: TTCATGGTTGGACCCTTCTC/TGTTTTCTACCAAGGAAATCCTGTAT. PCR products were treated with exonuclease I and shrimp alkaline phosphatase (New England Biolabs, Inc.) and Sanger sequenced at the ONPRC Molecular Technology Core, or alternatively, assessed for restriction fragment length polymorphisms using *HaeIII* (p.Leu415Pro) or *MseI* (p.Asp343Gly) restriction enzymes (New England Biolabs, Inc.), followed by agarose gel visualization for cleavage assessment. Genotyping analysis of the p.Asp343Gly site with this method used a derived cleaved amplified polymorphic sequence (dCAPS; Neff *et al*. 2002) alternative primer TYRP1_Ex5_R2: CAGTGCTTGGAAGTTGGTTTATTTA.

### Genetic analysis

Genome-wide association study (GWAS) analyses were performed using either GBS (ONPRC) or WGS (CPRC) data with PLINK (Chang *et al*. 2015) and GEMMA (Zhou and Stephens 2012) using the Mmul_10 reference genome (Warren *et al*. 2020). As data from each center were gathered and analyzed independently, separate analysis pipelines were utilized to identify genetic associations. GBS data were aligned using the Burrows–Wheeler Aligner (Li and Durbin 2010) and genotyped following best practice recommendations from the Broad Institute using the Genome Analysis Toolkit (McKenna *et al*. 2010; Van der Auwera *et al*. 2013). This resulted in an initial set of 816,824 autosomal, biallelic variants identified from 1,697 ONPRC individuals. Data were then filtered to remove variants with a minor allele frequency (MAF) < 0.01, variants and individuals with call rates below 90%, and those not in Hardy–Weinberg equilibrium (*P* < 1e−6). Variants in linkage equilibrium were pruned using a pairwise *r*^2^ threshold of 0.99999 within a 50 single nucleotide polymorphism (SNP) window shifted in 5 variant increments. The resulting 95,054 variants from 1,694 individuals were analyzed with GEMMA using a univariate linear mixed model, after adjusting for relatedness using the centered relatedness matrix option. Unadjusted likelihood ratio test *P*-values were used for analysis of genetic associations and generation of a Manhattan visualization plot.

WGS from 64 CPRC individuals was reference aligned and Parabricks 3.6.1 haplotype caller and joint genotype caller were used to genotype variants on the Arizona State University computing cluster. Variants were then filtered using BCFtools (Danecek *et al*. 2021) to include only variants with a missing genotype rate < 0.3, MAF > 0.15, and excess heterozygosity test scores > 1e−12. PLINK was used to remove variants with Mendelian violations and test the resulting 3,298,419 biallelic autosomal variants for phenotype association analysis with resulting *P*-values calculated from unadjusted asymptomatic chi-squared tests. Initially, we modeled the 8 golden monkeys as possessing a single phenotype and observed a significant association with multiple loci on chromosome 15. However, since further analysis demonstrated only 5 of the 8 animals being homozygous for the alternative alleles at these locations, we tested the hypothesis that there was a different genetic basis for the 3 remaining golden monkeys by reanalyzing the dataset with these being classified with a separate phenotype code.

To look at genetically linked variants, WGS variant calls and alignments were manually inspected to identify the boundaries of a shared Run of Homozygosity (RoH) conserved among individuals homozygous for each candidate variant. Variants within these boundaries were compared to the mGAP dataset to determine if they could be excluded as causative based on homozygosity in nongolden individuals. Remaining variants were inspected and mapped to the human genome (hg38) to determine if they fell within any predicted regulatory regions using the GeneHancer database (Fishilevich *et al*. 2017).

### Protein structural analysis of TYRP1

To visualize the affected protein regions of candidate variants in relation to reported pathogenic variants, a list of OCA3-related loci from previous human studies was downloaded from the Human Gene Mutation Database (Stenson *et al*. 2003). These mutations (Supplementary Table 1), along with candidates identified in this study, were mapped to the primary amino acid sequence and to the secondary and tertiary protein structure of human TYRP1. The ChimeraX molecular graphics program was used with the human TYRP1 structure downloaded from the Protein Data Bank (PDB; Pettersen *et al*. 2021; Lai *et al*. 2017; Berman *et al*. 2000) to model protein structure and affected amino acid position. We used the human protein structure as human and macaque amino acid sequences are 97% identical and no tertiary structure exists in the Protein Data Bank for the rhesus macaque TYRP1 protein.

### Retinal imaging

Retinal imaging of macaques at ONPRC was performed under anesthesia induced with Telazol (3.5–5.0 mg/kg intramuscular injection) and maintained with inhalant isoflurane (1–2%) vaporized in 100% oxygen. Eyes were fully dilated with 1% tropicamide and 2.5% phenylephrine eyedrops and fitted with plano contact lenses. Imaging modes included color fundus photography (Zeiss FF450, Oberkochen, Germany) and spectral domain optical coherence tomography (SD-OCT; Spectralis, Heidelberg Engineering, Inc., Carlsbad, CA). Corneal curvature and axial length were measured with an IOL Master (Carl Zeiss Meditec, Inc., Dublin, CA) to allow calculation of retinal distances in mm as well as degrees of visual angle. Four golden monkeys were imaged for this study and compared to 27 typical wild-type controls.

Macular OCT images were analyzed for total retinal and retinal layer thickness at 4 loci: the single point at the foveal center and the average across the central 1 mm, 1–3 mm, and 3–6 mm annuli. Quantified layers included total retina, inner retina (outer plexiform, inner nuclear, outer plexiform ganglion cell, and nerve fiber layers), outer nuclear layer (photoreceptor nuclei), and photoreceptor inner and outer segment layers (ISOS), as illustrated in Fig. 2b. Layer measurements were done with Spectralis segmentation software (EyeExplorer 1.9.10.1), with manual inspection and correction of each slice.

Foveal shape parameters were determined from horizontal OCT b-scans through the foveal center. Retinal thickness was measured at the foveal center (point of minimum thickness) and at the point of maximum thickness at the crest of the foveal slope, both nasal and temporal to the foveal center. Foveal width was calculated as the distance between the nasal and temporal foveal crests, while nasal and temporal foveal depths were calculated as the difference between the thickness of the foveal center and respective foveal crests.

## Results

### Characterization of golden rhesus macaque phenotype

**Fig. 1.**
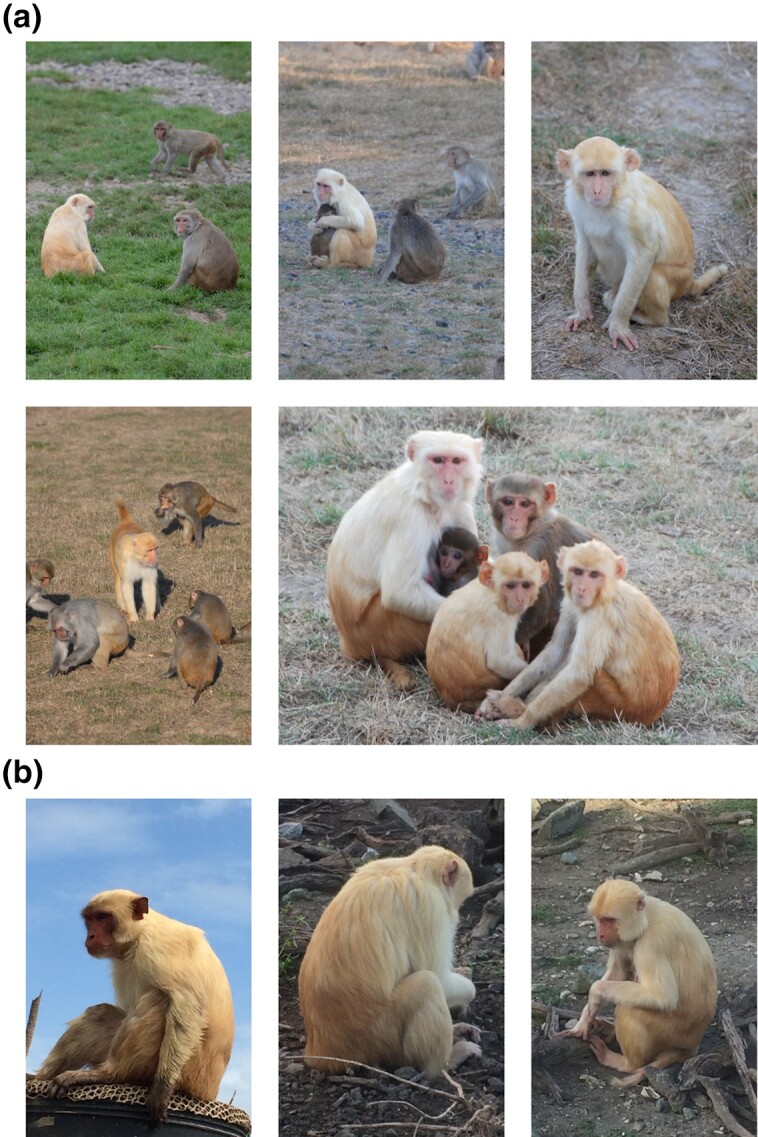
Characterization of the rhesus macaque golden phenotype. a) Photographs of typical golden animals alongside wild-type rhesus at ONPRC. b) CPRC golden monkeys include both those linked to *TYR* (left) and *TYRP1* (center and right). Photos in (a) by Kristine Coleman. Photos in (b) reused with permission from Marina M. Watowich.

Rhesus macaque coloration is typically bipartite, with a reddish agouti dorsal (lower back) pelage that is noticeably lighter than the fur on the upper back and extremities, which can vary from gray to brown and change with age and season (Fooden 2000). Bare and fur-covered skin is pale with the exception of the face and genital regions which vary from pink to red based on changes in blood flow and oxygenation of these regions for signaling functions (Dubuc *et al*. 2014; Higham *et al*. 2013). The golden phenotype in rhesus macaques is characterized by lighter-colored fur that remains hypopigmented from birth throughout the lifetime of the animal and has an estimated occurrence rate in the wild of 1/10,000 (Pickering and Van Wagenen 1969). The typically brown or gray fur has instead a pale yellowish color, while the dorsal region retains a reddish contrast as does the red skin coloration observed in the face and genitals. Since its initial description, monkeys with the golden phenotype have spontaneously appeared at both ONPRC (Fig. 1a) and CPRC (Fig. 1b). Some descriptions of the golden phenotype have described pale skin and blue eyes (Widdig *et al*. 2016). However, there was not an appreciable difference in skin pigmentation observed in our study, and changes in eye color were inconsistent or challenging to document without retinal imaging.

**Fig. 2.**
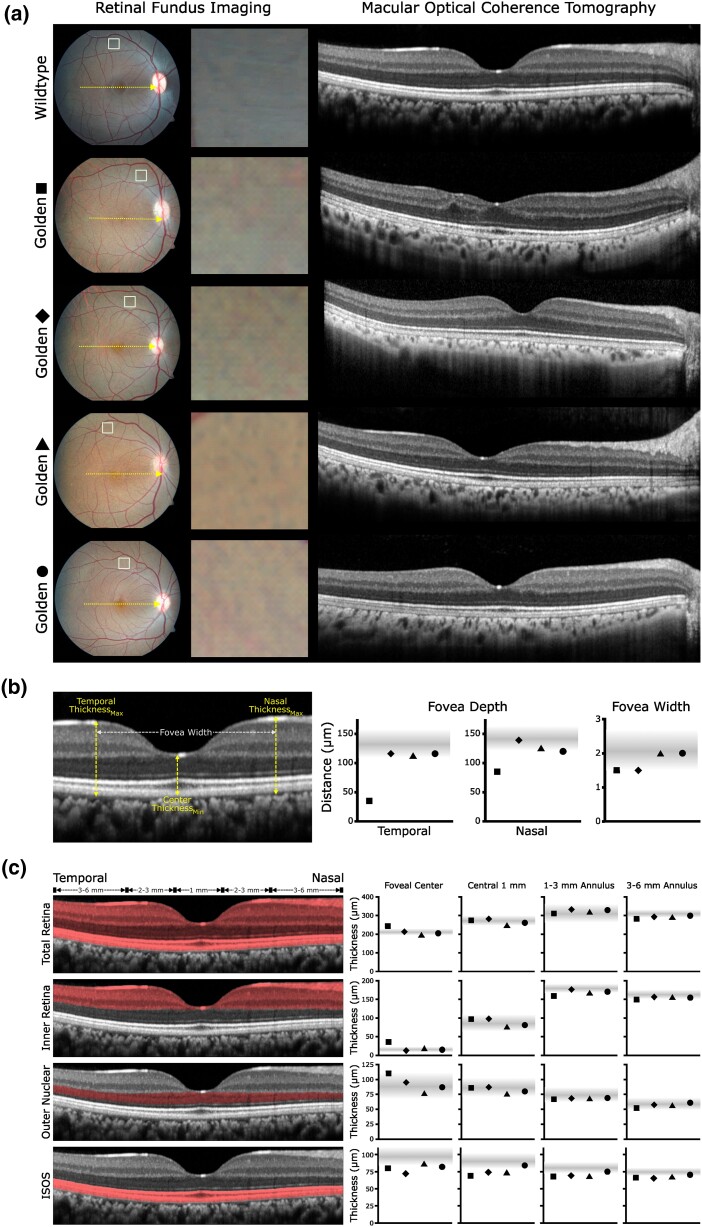
Retinal imaging of golden macaques. a) Color retinal fundus imaging of ONPRC macaques (left column) demonstrates characteristic hypopigmentation compared to a representative wild-type animal. The middle column shows magnified images of the indicated boxes at similar loci in the superior retina for further comparison. Macular horizontal OCT b-scans through the center of the fovea are shown on the right, illustrating foveal hypoplasia in 1 golden individual but normal foveal architecture in the other 3 cases. Genotypes of the goldens macaques are TYRP1 ^p.Leu415Pro/p.Leu415Pro^ (square and diamond), TYRP1 ^p.Asp343Gly/p.Leu415Pro^ (triangle), and TYRP1^p.Asp343Gly/p.Asp343Gly^ (circle). b) Measurements of foveal depth and width in the 4 golden individuals compared to wild-type animals. Foveal OCT b-scan on the left illustrates the location of nasal and temporal foveal crests and foveal center used for maximum and minimum thickness measurements and calculation of foveal depth and width shown on the right. Shaded area indicates range of values observed in normal wild-type controls (*n* = 27). c) Measurements of retinal layer thickness. Retinal layers used for corresponding measurements are highlighted. Layer thickness for 4 golden individuals is shown along with the range observed in wild-type controls (*n* = 27).

Color fundus imaging of 4 individuals at ONPRC was consistent with golden monkeys displaying an appreciable but variable degree of retinal hypopigmentation (Fig. 2a) compared to normal rhesus macaques. In 1 golden monkey, OCT imaging demonstrated notable incursion of the inner retinal layers and shallow foveal pit similar to grade 1 foveal hypoplasia observed in humans (Thomas *et al*. 2011). The other golden monkeys imaged showed normal foveal structure. The degree of foveal hypoplasia was verified by measurement of foveal depth (Fig. 2b; Supplementary Table 2), which was approximately 30% of the value of the other golden cases (35 µm vs. 116, 113, and 116 µm) and normal control animals (mean thickness of 130 µm). Foveal width in this individual and 1 other golden was also narrower than the observed range in control monkeys. Analysis of the thickness of retinal layers at concentric loci within the central retina (Fig. 2c) showed minor differences compared to the range observed in wild-type monkeys, with slight increases in total retinal, inner retina, and outer nuclear layer thicknesses at the foveal center for the case with foveal hypoplasia. There were also slight decreases in ISOS thickness across all loci for 3 of the 4 golden individuals.

**Fig. 3.**
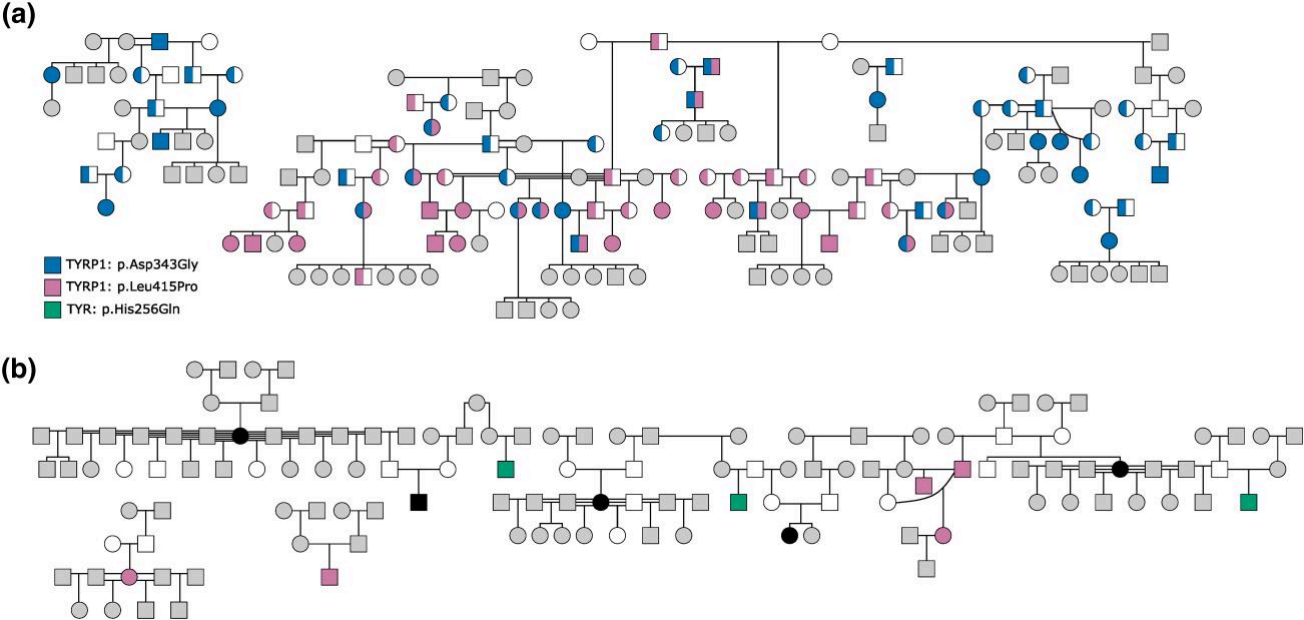
Inheritance pattern of golden rhesus macaque phenotype. a) Pedigree analysis of golden ONPRC macaques showing recessive single gene recessive inheritance pattern. Circles and squares (females and males, respectively) are filled based on *TYRP1* genotype as indicated. Solid and semifill indicate homozygous and heterozygous states for each allele. Unaffected individuals are shown as white if homozygous for wild-type *TYRP1* alleles or gray if not genotyped. b) Inheritance of the golden phenotype at CPRC is consistent with autosomal recessive trait inheritance. Golden macaques used in GWAS analysis are homozygous for *TYR*: p.His256Gln or *TYRP1*: p.Leu415Pro. White fill indicates wild-type family members used in GWAS analysis while gray (unaffected) and black (golden) were not genetically analyzed.

The first documented golden monkey on Cayo Santiago was born in 1972, and birth rate in subsequent years has been 8–52 times greater than what is estimated to occur in the wild, likely the result of a founder effect stemming from the small number of trapped monkeys used to populate the island (Kessler and Kaufman 1986; Widdig *et al*. 2016). A visual screen of the ONPRC rhesus colony identified 18 living members with an appearance consistent with the golden phenotype. Examination of the historical electronic health records identified another 36 individuals distinctly noted as golden, or having an appearance consistent with the description, resulting in a total of 54 recorded cases at ONPRC. The first case recorded was born in 1968. Pedigree analysis of affected individuals was consistent with an autosomal, recessive mode of inheritance at ONPRC (Fig. 3a) and CPRC (Fig. 3b).

### Independent GWAS analyses replicate region of interest on chromosome 15

**Fig. 4.**
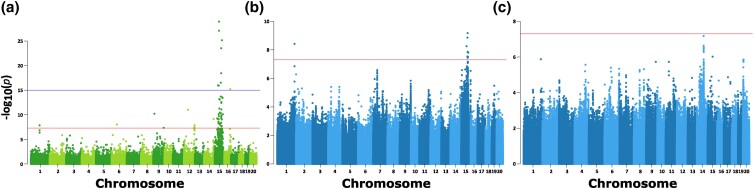
Genome-wide association of rhesus macaque golden phenotype. a) Manhattan plot of GWAS of GBS data from ONPRC colony (*n* = 1,694) showing marker loci associated with the golden phenotype on chromosome 15 with lines showing the significance thresholds of *P* < 5e−8 and 1e−15. b) WGS data from CPRC (*n* = 64) all demonstrate a phenotypic association with loci on chromosome 15. c) Reanalysis of the same data after accounting for 5 golden monkeys homozygous for *TYRP1* allele showing an association with loci on chromosome 14.

GBS is a high-throughput means of genotyping genetic variants, producing interspersed regions of deep sequence coverage and yielding a reliable set of genetic markers suitable for pedigree analysis or GWAS (Bimber *et al*. 2016). Markers with statistically significant associations with a specific trait can be investigated further with targeted sequencing of candidate genes in the surrounding genomic regions. GBS-based data from 1,694 ONPRC rhesus macaques, including 31 golden monkeys, were used to identify markers associated with the trait, revealing a strong association with specific variants on chromosome 15 (Fig. 4a). The often-used *P*-value threshold of 5e−8 yielded a total of 48 significant markers, while a more stringent threshold of 1e−15 resulted in just 9 significantly associated genetic markers, 8 of which were clustered on a stretch of chromosome 15 spanning ∼47 Mb (Supplementary Table 3). The other marker that reached this level of significance was located on chromosome 16 (16:70,595,597G > A). A search of genes known to affect pigmentation (Bakker *et al*. 2022) revealed the candidate gene *TYRP1* in the vicinity of associated markers (15:70,880,394–70,896,634). Closer analysis of the regions surrounding the markers on the other chromosomes that reached either of the 2 thresholds of significance did not identify any genes known to be linked to skin or hair pigmentation, nor any coding variants that segregated with phenotype inheritance. Additionally, these other markers were found in isolation and not surrounded by other significant variants, consistent with being false positives.

**Fig. 5.**
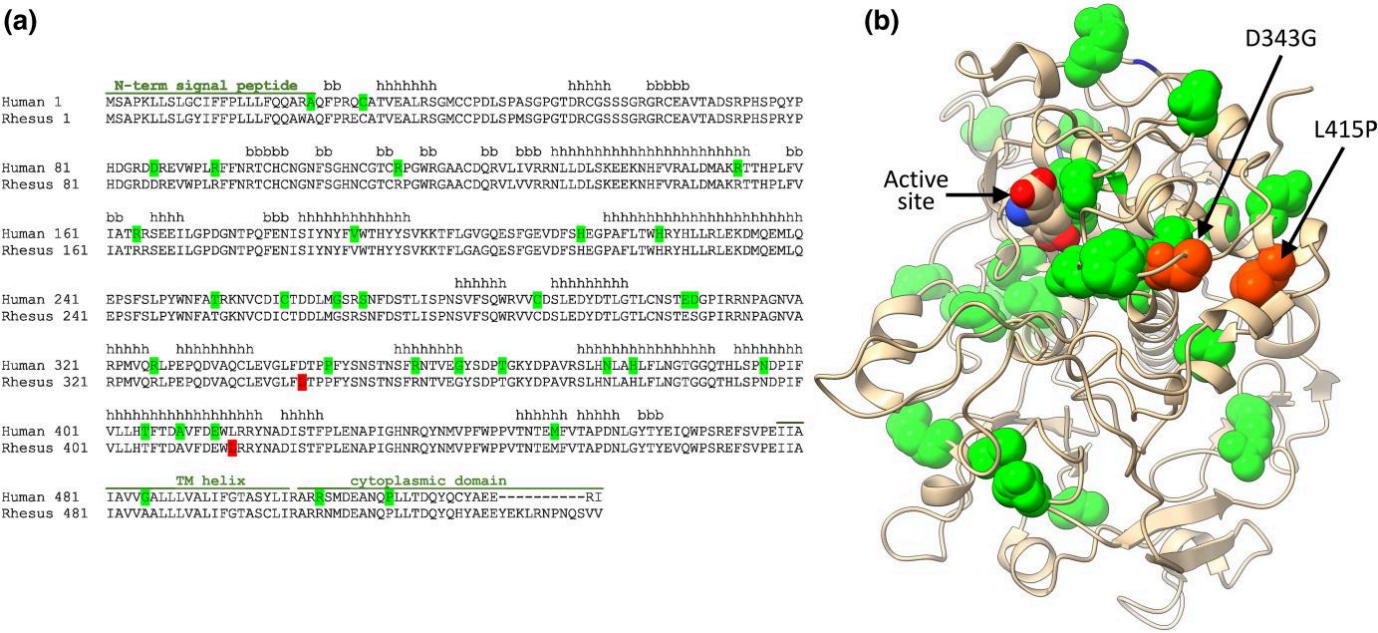
TYRP1 structural modeling of candidate variant locations. a) Primary and secondary structures of the TYRP1 protein. Highlighted amino acids in the human protein sequence indicate locations of mutations known to disrupt TYRP1 function and are associated with blonde hair, OCA3, or albinism in humans. Amino acids highlighted in the rhesus macaque sequence indicate the locations of the 2 missense mutations uncovered in this study that are associated with golden phenotype in rhesus macaques. Above the primary structure, the corresponding secondary structure elements are shown. b) The tertiary structure of the human TYRP1 protein (PDB ID: 5m8n). The amino acid residues highlighted in (a) are shown along with arrows showing the location of the 2 macaque mutations. Note that the 2 macaque sites are located near the majority of human mutation sites and the TYRP1 active site.

The GBS-based GWAS analysis identified markers suggesting that a region on chromosome 15 was associated with the golden phenotype but did not provide sequencing coverage of specific genes or variants. For this purpose, we made use of the mGAP data resource to retrieve genome-wide variant information on rhesus macaques residing at the ONPRC, CPRC, and other primate research centers. A survey of all previously observed coding variants in rhesus *TYRP1* identified 33 missense variants, 1 early termination, and 2 in-frame insertions. However, only 8 of these variants had been observed in monkeys from ONPRC (Supplementary Table 4). Of the 8 previously observed ONPRC missense variants, 6 were exceedingly rare (<5 carriers identified out of 951 genotyped individuals) and unlikely to account for the prevalence of the observed trait. Furthermore, pedigree and demographic data showed that none of these 6 variants were carried by known golden monkeys or obligate carriers, ruling them out as putative causative variants. The remaining 2 variants, (c.1,028A > G; p.Asp343Gly) and (c.1,244T > C; p.Leu415Pro) were selected for further pedigree analysis as potential causal variants. Both alter amino acids that are highly conserved (Fig. 5) throughout mammal and vertebrate species and generate amino acid substitutions predicted to be deleterious based on variant effect predictors (SIFT = 0; 0; PolyPhen = 1; 0.968; CADD = 28.9; 30; for p.Asp343Gly and p.Leu415Pro, respectively). Their observed allele frequencies in the mGAP data set, as well as in the ONPRC-specific cohort (0.025; 0.01 and 0.037; 0.019) approximate Hardy–Weinberg equilibrium and are consistent with the ONPRC golden occurrence rate of 1:1,333 (MAF = 0.027) or the rate of 1:10,000 (MAF = .01) estimated to occur in the wild (Pickering and Van Wagenen 1969).

Based on the pedigree analysis and prevalence of the 2 candidate *TYRP1* variants, we hypothesized that the ONPRC golden phenotype was caused by homozygous or compound heterozygous genotypes of either p.Asp343Gly or p.Leu415Pro missense variants. The 2 alleles are separated by less than 2,500 bp and the observed inheritance pattern is consistent with them residing on separate haplotypes. Accordingly, an extensive pedigree screen of all ONPRC golden macaques with available genetic material targeting the 2 alleles (Fig. 3a) confirmed ONPRC golden monkeys carry 1 of the 3 genotype possibilities consistent with a compound recessive trait (TYRP1^p.Asp343Gly/p.Asp343Gly^, TYRP1^p.Leu415Pro/p.Leu415Pro^, TYRP1^p.Asp343Gly/p.Leu415Pro^; *n* = 13; 12; 11 respectively). A full analysis of the sequences deposited in the mGAP database, in combination with the pedigree screen (*N* > 3,000 total animals with genotype information at these loci) revealed only 2 macaques that were predicted to be golden based on genotype but were not identified as such in the initial demographic search. Both were deceased and unable to be visually examined. The health records for these individuals did not include information on fur coloration and both monkeys had significant confounding factors that potentially explain why altered fur pigmentation was not noted (e.g. premature birth with severe jaundice and alopecia). Thus, it was not possible to determine if these individuals supported or were in conflict with the phenotypic association.

An independent genetic analysis was performed on macaques located on Cayo Santiago at the CPRC. This analysis included 8 golden monkeys, 19 primary family members, and 37 unrelated wild-type monkeys. GWAS analysis revealed a cluster of trait-associated variants on chromosome 15 (Fig. 4b; Supplementary Table 5) suggesting the possibility of an identical or related genetic basis for the golden phenotype observed at both centers. The mGAP database includes 2 cataloged missense variants in the *TYRP1* gene from CPRC monkeys (Supplementary Table 4), and the WGS data from the 64 individuals included in this study did not include novel *TYRP1* coding variants. The p.Leu415Pro candidate variant was observed in the CPRC cohort which partially recapitulated the association findings from ONPRC, with 5 of the 8 golden monkeys having the TYRP1^p.Leu415Pro/p.Leu415Pro^ genotype. Surprisingly, the other 3 golden monkeys in this group were not homozygous for any *TYRP1* coding variants, and no CPRC macaques carried the p.Asp343Gly variant.

We hypothesized that the remaining 3 cases were linked to a second genetic locus. Therefore, we reanalyzed the CPRC dataset with the 3 unexplained cases coded as having a distinct “phenotype.” This analysis revealed an association with variants on chromosome 14 which did not appear in the first analysis and was centered on the *TYR* gene (Fig. 4c; Supplementary Table 6). The lower statistical association observed with these variants (6 variants below *P* < 5e−7) is likely a result of having a smaller group representing the alternative phenotype in the analysis. A genome-wide screen for exonic variants fitting the predicted recessive inheritance criteria of being homozygous in the 3 remaining cases, and wildtype or heterozygous in the group of unaffected individuals was performed and yielded a total of 3 candidate variants. Two of these variants were synonymous mutations (1 in *SULF1* and 1 in *TYR*) and not predicted to affect protein function. Additionally, according to the mGAP database, both variants have been observed in the homozygous state in several nongolden animals. Together, this suggested that they were unlikely to be causative. In the mGAP database, no homozygous individuals had been reported for the third variant, (*TYR* c.768C > G; p.His256Gln) while 9 monkeys had been genotyped as carriers (MAF = 0.002). While variant prediction algorithms predicted only mild effects from the substitution (SIFT = 0.05; PolyPhen = 0.533; CADD = 8.802), the variant affects the same amino acid as a known pathogenic human allele linked to OCA1 (*TYR* p.His256Tyr; rs61754370; Zahed *et al*. 2005; Camand *et al*. 2001) and was postulated to be the third causal variant of the golden phenotype in rhesus.

The potential influence of linked variants in other genes or regulatory regions was explored by comparing regions of conserved runs of homozygosity in golden monkeys with WGS data. The 3 candidate variants are inherited in conserved haplotype blocks that encompass several surrounding genes (Supplementary Table 7). Most variants found on these inherited haplotypes could be ruled out as causative as they are frequently observed in nongolden rhesus. However, there were several variants that matched the observed inheritance pattern of the candidate variants and could not be excluded based on inheritance pattern. While many of these variants are intergenic and not predicted to impact protein coding, a few reside in regulatory domains (as classified by GeneHancer) and thus could potentially influence the expression of genes in these areas and contribute to the golden phenotype.

### Predicted protein structural changes caused by TYRP1 variants

We used known protein structure information to provide insight into how the candidate golden variants might be affecting protein function. First, we mapped the putatively causal loci identified in rhesus macaques to the primary amino acid structure, and for the TYRP1 loci, to the secondary, and tertiary human protein structures. The Leu415Pro mutation was not only in the same helix formation as 4 known human albinism-associated mutations but introduction of prolines into helices typically prevents helix formation, thus the Leu415Pro mutant may disrupt the protein structure or folding (Chou and Fasman 1978). Strikingly, in the tertiary structure, both TYRP1 mutations are located close together and near the protein active site (Fig. 5b). Additionally, these residues are found in a spatial region that contains a large number of known albinism-associated human mutations, suggesting that this region of the TYRP1 protein may be easily disrupted by mutations. Functionally, the mechanistic link between TYRP1 structural disruption and pigmentation color remains unknown in humans. Previous studies have suggested that albinism-associated mutations may disrupt TYRP1 catalytic activity, interactions between TYRP1 and TYR, or intracellular transport of TYRP1. As noted, the missense variant identified in *TYR* alters an amino acid (His256) known to be associated with OCA1 (Camand *et al*. 2001; Opitz *et al*. 2004; Zahed *et al*. 2005) and previous modeling studies highlighted this amino acid as 1 of 24 contact residues predicted to be at the TYR-TYRP1 binding interface (Lavinda *et al*. 2021).

## Discussion

We used independent GWAS analysis of 2 rhesus macaque populations to attribute the genetic basis for the golden rhesus macaque phenotype to variants in the *TYRP1* and *TYR* genes. Appropriately, both genes function in the melanogenesis pathway and have been identified as the basis for various color traits in species across the animal kingdom. To our knowledge, this is the first reported example of *TYRP1*-linked pigmentation variation in an NHP, and the *TYR* variant identified is genetically novel from those previously characterized in other rhesus macaques (Ding *et al*. 2000; Wu *et al*. 2020). This lighter coloration morph appears benign in the studied populations, as golden monkeys are typically healthy and do not exhibit any overt deficits. However, neither population experiences the pressures or threats of living in the wild. Nevertheless, the free-ranging structure of the Cayo Santiago macaque population provides a unique setting allowing for the effect of the color pelage on social interactions, hierarchy, and sexual selection to be specifically studied.

Mutations to melanogenesis-related genes have been identified as drivers for color traits in many animals, and often these traits are considered to be either benign or have advantageous qualities. Conversely, in humans, loss-of-function mutations in these same genes have been linked to OCA where the loss of melanin in skin and retina can have negative health consequences. Differences in melanocyte localization and activity drive coloration differences across animals, and consequently, genetically related perturbations to this pathway result in species-specific phenotypic differences. Most mammalian species, and primates specifically, express melanocytes deep in the dermis and hair follicles, which results in colored fur covering skin that is generally unpigmented (Fernández *et al*. 2021). In contrast, humans have lost the majority of fur covering and instead rely on melanocytes for adaptive skin pigmentation (Jablonski and Chaplin 2017). This dichotomy was recently highlighted by the American black bear cinnamon morph being linked to a loss-of-function variant in *TYRP1* that is genetically identical to a human variant classified as a pathogenic cause of OCA (Puckett *et al*. 2022). In an analogous manner, we predict that on a genetic and biochemical level, the mechanism responsible for the golden pelage in rhesus mimics aspects of OCA without resulting adverse health effects associated with exposed hypopigmented skin. The molecular conservation of disrupting the melanogenesis pathway in an NHP without resulting detrimental health effects could be utilized for gene therapy, pharmaceutical intervention, or mechanistic studies without the need to generate or sustain an NHP model requiring specialized care and treatment.

Ocular imaging of golden macaques at ONPRC showed retinas that were hypopigmented compared to controls and had foveae that ranged from normal to hypoplastic. This variation is consistent with a previous study of Cayo Santiago macaques that showed a spectrum of foveal hypopigmentation levels in golden macaques and an absence of ocular instability (Dawson *et al*. 2004). Similarly, we do not observe nystagmus or photophobia in golden monkeys nor the consistent absence of fovea development that was recently observed in a report of *TYR* and *OCA2*-linked albino rhesus (Wu *et al*. 2020). It is interesting that the 2 genetically distinct golden phenotypes appear so similar despite disrupting proteins that have distinct roles in the melanogenesis pathway. TYR is the only enzyme strictly required for melanogenesis, as it alone catalyzes the initial breakdown of L-tyrosine, but the exact mechanism of TYRP1 activity remains elusive, despite its structural similarity to TYR and vast abundance in melanocytes (Tai *et al*. 1983; Vijayasaradhi *et al*. 1990). Instead of having a related enzymatic role, it has been proposed that TYRP1 may function to stabilize TYR (Kobayashi *et al*. 1998) though the formation of stable heterocomplexes (Lavinda *et al*. 2021) or other mechanisms (Dolinska *et al*. 2019), and specifically promote the synthesis of the darker eumelanin pigment. With this context, the golden phenotype is consistent with eumelanin-specific inhibition which would result from TYRP1 disruption, as the affected areas are those that typically display darker fur color while areas that are typically light and pheomelanin rich like the rump are unaffected. Similarly, the specific TYR amino acid substation identified in these monkeys is predicted to occur on the TYR-TYRP1 binding interface (Lavinda *et al*. 2021), so a similar phenotype could be explained by disrupting the ability of TYRP1 to interact with TYR.

Advances in genomic research have yielded tools for unprecedented population-level study of rhesus macaques. The mGAP database features whole-genome and whole-exome sequences for rhesus macaques from multiple primate centers (3,455 subjects from 8 National Institutes of Health (NIH)-supported primate centers, mGAP 2.4, April 28, 2023 release date). For the identification of pathogenic variants of observed phenotypes, the depth of known and observed variant allele frequencies is vital, as it allows for the exclusion of countless variants without requiring a high number of affected subjects or a complete pedigree analysis. In this study, we were able to use a genome-wide survey of allele frequencies to narrow the list of candidate gene variants to conclusively determine the causative variants for the golden phenotype. The same approach has been used for the successful identification of other novel rare disease models in macaques (Peterson *et al*. 2019; Johnson *et al*. 2020; Sherman *et al*. 2021) and will likely uncover additional NHP genetic models in the future.

## Supplementary Material

jkad168_Supplementary_Data

## Data Availability

Data on genetic variants can be accessed through mGAP (https://mgap.ohsu.edu/), which also includes links to deposited raw sequence for all individuals in the NCBI Sequence Read Archive (http://www.ncbi.nlm.nih.gov/sra). Sequence Read Archive (SRA) accessions and mGAP IDs for GBS samples used for GWAS are provided in Supplementary Table 8. Sequence information for ONPRC golden monkeys in mGAP is listed in Supplementary Table 9, and WGS data used for the analysis of Cayo Santiago monkeys are detailed in Supplementary Table 10. Supplemental tables and data used in GWAS analysis have been uploaded to the Genetics Society of America Figshare repository: https://doi.org/10.6084/m9.figshare.23706093.
