# Supplementary material for: Genetic variants in melanogenesis proteins TYRP1 and TYR are associated with the golden rhesus macaque phenotype
Source: G3 (Bethesda). 2023 Jul 31;13(10):jkad168. doi: 10.1093/g3journal/jkad168 (PMC10542561; doi:10.1093/g3journal/jkad168)
Supplement: jkad168_Supplementary_Data [file jkad168_supplementary_data.zip › SupplementalInfo20230710.pdf]

Supplemental information for ‘Genetic variants in melanogenesis proteins *TYRP1* and *TYR* are associated with the golden rhesus macaque phenotype’

SUPPORTING TABLES

Supplemental tables 1-10 (Tables S1-10) are included in attached file (STables_Peterson_etal.xlsx)

Table S1: List of variants classified as pathogenic in the Human Gene Mutation Database (Stenson et al. 2003) highlighted in TYRP1 protein modeling
Pathogenic *TYRP1* variants classified as pathogenic used for Retinal measurements from ONPRC golden and wildtype rhesus macaques.
Table S2: Retinal measurements from ONPRC golden and wildtype rhesus macaques.
Table S3: Summary information for variants significantly (p<5e-8) associated with golden phenotype in ONPRC macaques. P-values used for generation of Manhattan plot (Fig. 4a) were calculated with GEMMA (v0.98.5)(Zhou and Stephens 2012) using the unadjusted likelihood ratio test (GEMMA_p_lrt) from a univariate linear mixed model. Other statistical test score parameters are included including Odds Ratios and p-values from an association analysis with PLINK(v1.90b6.26)(Chang et al. 2015).
Table S4. *TYRP1* coding variants previously identified in ONPRC and CPRC cohorts along with observed allele frequencies.
Table S5. Summary information of variants in CPRC rhesus macaques significantly associated (p<5e-8) with the golden phenotype. P-values were calculated with PLINK using an unadjusted asymptomatic chi-squared test (Fig 4b).
Table S6. Summary information of SNPs identified as associated (p<5e-7) with the golden phenotype in reanalysis of CPRC WGS data after accounting for *TYRP1* based golden rhesus (Fig. 4c). The lower significance threshold was used since less subjects with the target phenotype were in the analysis.
Table S7: Variants within conserved regions of homozygosity in golden rhesus including gene location and overlapping regulatory regions predicted with GeneHancer(Fishilevich et al. 2017).
Table S8: Information regarding GBS data used for ONPRC GWAS analysis of GBS data, including mGAP(Bimber et al. 2019) (https://mgap.ohsu.edu/) Subject IDs, phenotype status, and SRA accession numbers.
Table S9: Golden rhesus macaque identities with WGS data deposited in mGAP.
Table S10: Raw sequence information for WGS data used in the GWAS analysis of the golden phenotype at CPRC, including SRA accession numbers.

SUPPORTING FILES

A zip archive file (SFiles_Peterson_etal.zip) is included with data and scripts used for GWAS analysis. Variant calls for each data set are supplied in PLINK format (note that the two CPRC analysis only differ in coded phenotype).

Bimber, B.N., M.Y. Yan, S.M. Peterson, and B. Ferguson, 2019 mGAP: the macaque genotype and phenotype resource, a framework for accessing and interpreting macaque variant data, and identifying new models of human disease. *BMC genomics* 20 (1):176.
Chang, C.C., C.C. Chow, L.C. Tellier, S. Vattikuti, S.M. Purcell *et al.*, 2015 Second-generation PLINK: rising to the challenge of larger and richer datasets. *GigaScience* 4 (1).
Fishilevich, S., R. Nudel, N. Rappaport, R. Hadar, I. Plaschkes *et al.*, 2017 GeneHancer: genome-wide integration of enhancers and target genes in GeneCards. *Database (Oxford)* 2017.
Stenson, P.D., E.V. Ball, M. Mort, A.D. Phillips, J.A. Shiel *et al.*, 2003 Human Gene Mutation Database (HGMD): 2003 update. *Hum Mutat* 21 (6):577-581.

Zhou, X., and M. Stephens, 2012 Genome-wide efficient mixed-model analysis for association studies. *Nature Genetics* 44 (7):821-824.
